# PLGA Implants for Controlled Drug Delivery and Regenerative Medicine: Advances, Challenges, and Clinical Potential

**DOI:** 10.3390/ph18050631

**Published:** 2025-04-27

**Authors:** Hossein Omidian, Renae L. Wilson

**Affiliations:** Barry and Judy Silverman College of Pharmacy, Nova Southeastern University, Fort Lauderdale, FL 33328, USA; rw1273@mynsu.nova.edu

**Keywords:** PLGA implants, controlled drug delivery, regenerative medicine, personalized implants, biocompatible polymers, 3D printing, in situ forming systems, predictive modeling

## Abstract

Poly(lactide-*co*-glycolide) (PLGA) implants have become a cornerstone in drug delivery and regenerative medicine due to their biocompatibility, tunable degradation, and capacity for sustained, localized therapeutic release. Recent innovations in polymer design, fabrication methods, and functional modifications have expanded their utility across diverse clinical domains, including oncology, neurology, orthopedics, and ophthalmology. This review provides a comprehensive analysis of PLGA implant properties, fabrication strategies, and biomedical applications, while addressing key challenges such as burst release, incomplete drug release, manufacturing complexity, and inflammatory responses. Emerging solutions—such as 3D printing, in situ forming systems, predictive modeling, and patient-specific customization—are improving implant performance and clinical translation. Emphasis is placed on scalable production, long-term biocompatibility, and personalized design to support the next generation of precision therapeutics.

## 1. Introduction

PLGA is a pivotal material in biomedical engineering, extensively utilized for its biocompatibility, tunable degradation, and adaptability in drug delivery and tissue engineering. As an FDA-approved polymer, PLGA facilitates sustained and controlled drug release through implants that degrade into lactic acid and glycolic acid—non-toxic byproducts naturally metabolized by the body [1,2,3]. Its versatility, governed by variations in the lactic-to-glycolic acid ratio, molecular weight, and structural modifications, enables the design of implants tailored for diverse therapeutic domains including oncology, endocrinology, orthopedics, and neurology [4,5,6,7].

Critical implant characteristics such as degradation kinetics, drug release profiles, and mechanical performance are modulated through the polymer composition and incorporation of functional additives. Modifications with poly(ethylene glycol) (PEG), poloxamer, and surfactants improve biocompatibility, reduce burst release, and fine-tune release behavior [2,8,9,10,11,12]. These advancements have enabled prolonged drug delivery over periods ranging from days to several months, supporting applications in chronic disease management, localized chemotherapy, and regenerative therapies [6,13,14].

Despite these benefits, PLGA-based implants encounter key limitations. The initial burst release can compromise therapeutic efficacy, especially in sensitive systems such as ocular and neural delivery routes [15,16]. The acidic microenvironment produced during degradation can destabilize labile biomolecules, while high manufacturing costs and technical complexities hinder large-scale production [17,18,19]. Additionally, variability in polymer composition and environmental conditions may lead to inconsistent release profiles, highlighting the need for stringent quality control and predictive modeling to ensure clinical reliability [12,20,21].

This review presents a comprehensive evaluation of PLGA-based implants, emphasizing material characteristics, fabrication strategies, and application-specific progress. It further addresses prevailing challenges and discusses emerging directions aimed at optimizing PLGA systems for improved therapeutic performance. By integrating interdisciplinary insights, this work seeks to foster continued innovation and collaboration across biomedical fields.

## 2. Essential Materials for PLGA Implant Design

The versatility and efficacy of PLGA-based implants derive from the precise engineering of their constituent materials, including the polymer matrix, therapeutic agents, functional additives, and structural components. Strategic selection and integration of these materials enable the optimization of drug release kinetics, biocompatibility, and mechanical performance across a broad range of biomedical applications.

### 2.1. PLGA as a Base Polymer

PLGA is a leading biodegradable polymer for implantable systems due to its tunable degradation rate and drug release profile. These properties can be tailored by adjusting the lactic-to-glycolic acid ratio (e.g., 50:50 or 75:25) and molecular weight (15–53 kDa) [1,5,22,23,24,25,26,27,28,29]. A 50:50 ratio typically results in faster degradation and earlier drug release due to higher hydrophilicity and rapid water uptake, making it suitable for applications requiring quick therapeutic onset, such as oncology or anti-infective therapies [22,26,27]. In contrast, a 75:25 ratio slows water ingress and polymer erosion, enabling more prolonged and sustained release, which is beneficial in chronic indications like hepatitis B or ocular delivery [1,5,30].

Further control over degradation kinetics and drug release behavior is achieved through end-group modifications, such as acid and ester end-capping, which influence surface roughness, mechanical strength, and early release rates [21,30]. The LA:GA ratio also affects implant morphology; faster-degrading 50:50 PLGA generates porosity more quickly and lowers local pH through acidic byproducts, which may be suboptimal for sensitive tissues like the inner ear [21]. In such cases, formulations using higher lactic content or PEG-PLGA blends help delay degradation and stabilize the microenvironment [21,24]. Blending PLGA with other biodegradable polymers like polylactic acid (PLA) or polycaprolactone (PCL) enhances mechanical strength and flexibility, broadening its applicability in drug delivery and regenerative medicine [31,32].

### 2.2. Therapeutic Agents Delivered via PLGA

PLGA-based implants support controlled delivery of a wide spectrum of therapeutic agents. For inflammation-associated disorders, dexamethasone offers prolonged release in ocular and cochlear applications [16,23,33]. Anticancer agents such as doxorubicin, paclitaxel, and cisplatin are incorporated into PLGA systems for localized delivery in cancers, including gliomas and breast cancer [6,13,26,27]. In regenerative medicine, growth factors like basic fibroblast growth factor (bFGF), recombinant human bone morphogenetic protein-2 (rhBMP-2), and vascular endothelial growth factor (VEGF) facilitate angiogenesis, neurogenesis, and osteogenesis when delivered through PLGA-based scaffolds and microspheres [34,35,36]. Additionally, PLGA serves as a delivery matrix for antibiotics, such as amoxicillin and vancomycin, in dental and ocular infections [29,37] and antiangiogenic agents like lupeol and corosolic acid in treating diabetic retinopathy and macular degeneration [38,39].

### 2.3. Additives, Nanocarriers, and Structural Enhancements

To optimize drug encapsulation, stability, and release profiles, various additives and structural elements are employed. PEG and acetyltributyl citrate (ATBC) are used to regulate flexibility, swelling, and diffusion, thereby improving the overall implant performance [33,40,41]. Stabilizers such as trehalose and beta-cyclodextrin (β-CD) enhance protein stability and hydrophilicity, supporting the delivery of sensitive biomolecules, including monoclonal antibodies [12,42]. In infection-prone environments, antibacterial agents like nanosilver and copper–selenium nanoparticles are incorporated to bolster antimicrobial activity and prolong implant function [43,44,45].

PLGA-based delivery platforms also utilize nanoparticles, microspheres, and scaffolds to achieve localized delivery and regenerative outcomes. Gold nanoparticles conjugated with antagomiR204, for example, enhance osteogenesis in diabetic patients [46]. Similarly, chitosan-based nanoparticles and PLGA microspheres loaded with doxorubicin or exendin-4 improve encapsulation efficiency and therapeutic outcomes in breast cancer and type 2 diabetes mellitus (T2DM)-associated dental applications [47,48].

Scaffolds integrating β-tricalcium phosphate (β-TCP) or hydroxyapatite (HA) with PLGA improve bone regeneration and osseointegration, key for orthopedic applications [32,49]. For cartilage and soft tissue repair, PLGA scaffolds combined with fibrin gels or human embryonic stem cells demonstrate enhanced mechanical stability and regenerative potential [50,51,52].

### 2.4. Solvents and Processing Aids

The successful fabrication of PLGA implants depends on the use of suitable solvents and processing aids. Hydrophilic solvents such as N-methyl-pyrrolidone (NMP) and glycofurol are essential in in situ forming systems for creating controlled-release matrices [23,53,54]. Dimethyl sulfoxide (DMSO) facilitates rapid solidification in injectable formulations, while agents like triacetin and ethyl heptanoate adjust viscosity and mitigate burst release, contributing to a more predictable and sustained drug release profile [55,56].

Table 1 examines the detailed composition of PLGA-based implants, including additives like PEG, PVA, and excipients. It links composition choices to their effects on implant properties, degradation, and release kinetics. Strategic integration of additives within PLGA systems allows for precise control over key characteristics, such as mechanical stability, biocompatibility, and drug release profiles. Excipients like PEG improve hydrophilicity, promoting uniform drug release, while surfactants reduce burst effects, ensuring steady delivery. Antimicrobial agents enhance biocompatibility and implant sterilization potential. The choice of lactide-to-glycolide ratios influences degradation times, allowing formulations to be customized for short-term or extended therapies. This table highlights how material design in PLGA implants enables the creation of safe, stable, and effective systems, supporting a range of therapeutic applications.

## 3. Key Properties of PLGA Implants for Medical Applications

PLGA-based implants are characterized by their tunable material and structural properties, enabling precise control over drug release, mechanical performance, and biocompatibility. These implants are engineered by integrating therapeutic agents, functional additives, and design strategies to meet specific clinical needs. Core attributes such as release kinetics, porosity, degradation behavior, and functional integration are tailored to optimize therapeutic outcomes across diverse medical applications.

### 3.1. Controlled Release Kinetics

Sustained drug release over extended durations is a hallmark of PLGA implants, reducing administration frequency while enhancing treatment efficacy. Rilpivirine-loaded systems sustain HIV therapy for 42 days [2], and ciprofloxacin hydrochloride implants maintain therapeutic levels for up to 65 days [82]. Drug release profiles range from biphasic to triphasic, accommodating both small molecules like ibuprofen and proteins such as cytochrome C [25,83]. Additives, including albumin–oleic acid conjugates (AOC) [62] and PEG [3], minimize burst release and promote near-zero-order kinetics, as observed in paclitaxel-loaded microspheres. Additionally, shape-controlled implants enhance release uniformity by maintaining consistent surface-to-volume ratios [84].

### 3.2. Porosity and Morphology

Implant porosity and surface morphology critically influence drug diffusion and tissue compatibility. Porous structures facilitate water uptake and degradation, accelerating drug release, as demonstrated in hydrogel–PLGA systems [85]. Stabilizing agents like MPEG and shellac protect labile proteins and peptides while maintaining controlled release [11,86]. Shellac functions as a pH-responsive barrier, shielding acid-labile biomolecules from the acidic microenvironment created by PLGA degradation and enabling delayed, more complete release once the matrix pH rises [11]. PEG, particularly in MPEG-PLGA diblock copolymers, enhances water absorption and matrix swelling, improving diffusion and uniform degradation, which reduces protein aggregation and supports sustained release [86]. In contrast, denser morphologies prolong degradation, supporting long-term drug delivery for conditions such as infections, prostate enlargement, and substance use disorders [57,61,87]. In situ forming implants (ISFIs) employ phase inversion to dynamically regulate porosity and control release kinetics [9].

### 3.3. Swelling and Degradation Behavior

**Figure 1:**
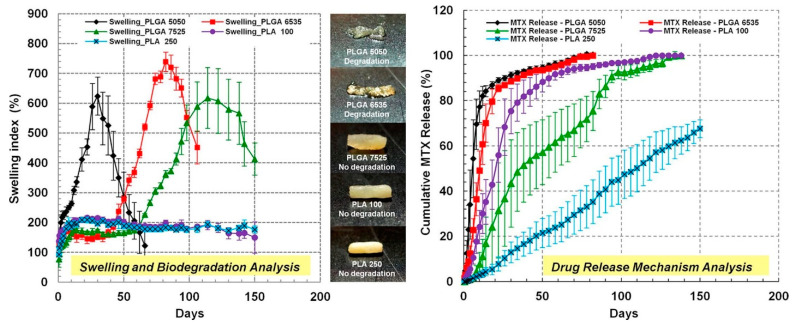
The swelling index and cumulative MTX drug release from PLGA/PLA-coated chitosan-based micro-implants. The figure shows pictures of different combinations of PLGA/PLA-coated chitosan–MTX micro-implants: PLGA 5050, PLGA 6535, PLGA 7525, PLA 100, and PLA 250. These implants provided MTX release for up to 5 months and a delayed swelling and biodegradation of the micro-implants. Adopted with permission [76].

Swelling and biodegradation directly impact drug release rates and mechanical integrity. PLGA systems can swell by up to 1700%, facilitating controlled diffusion of encapsulated agents [22]. The customization of lactic/glycolic content, molecular weight, and environmental pH allows precise modulation of degradation. For example, leuprolide acetate implants utilize closed-pore architectures to regulate hormonal therapy [88]. As shown in Figure 1, methotrexate (MTX) release from PLGA/PLA-coated chitosan implants correlates with structural swelling, underlining the importance of morphology in drug delivery performance.

### 3.4. Thermal and Mechanical Stability

Thermal and mechanical resilience is essential for the functionality and durability of PLGA implants during processing and implantation. The incorporation of hydroxyapatite (HA) or β-tricalcium phosphate (β-TCP) significantly improves mechanical strength, elasticity, and environmental durability—key requirements for orthopedic applications [82]. For instance, HA-grafted PLGA formulations demonstrate more than double the tensile strength of unmodified PLGA, and β-TCP/PLGA scaffolds maintain structural integrity in wet environments, supporting guided bone repair [32,49].

### 3.5. Biocompatibility and Reduced Inflammation

Biocompatibility is a critical determinant of implant success. PLGA systems are engineered to reduce immune responses and promote tissue integration. Scaffolds composed of small intestinal submucosa (SIS) exhibit reduced inflammation compared to conventional PLGA materials [50]. In neural applications, PLGA coatings mitigate glial scar formation, enhancing implant–tissue compatibility [89]. Furthermore, PLGA-coated titanium and ZrO_2_ implants improve osteoblast proliferation and antibacterial performance, validating their utility in dental and orthopedic settings [90,91].

### 3.6. Multifunctionality and Enhanced Therapeutic Outcomes

PLGA-based implants increasingly serve multifunctional roles, delivering multiple therapeutic benefits beyond controlled release. Dual drug–particulate systems enable concurrent delivery of vaccines and therapeutics, improving efficacy in immunological and infectious disease contexts [92]. MRI-visible implants support real-time tracking of degradation and drug release, advancing precision diagnostics [93]. Antiangiogenic systems delivering lupeol or corosolic acid show comparable efficacy to Bevacizumab in retinal disease models [38,39]. Hybrid PLGA–gelatin systems target lymphatic pathways, offering improved delivery for metastatic conditions [73].

The therapeutic utility of PLGA systems extends across numerous clinical applications. Curcumin-loaded formulations demonstrate anti-inflammatory and survival benefits in cancer models [31], while titanium implants coated with testosterone–alendronate enhance bone integration [94]. In diabetic bone regeneration, bFGF-loaded microspheres improve osteogenesis and implant contact [36]. Optimal pore sizes (300–500 µm) in PLGA scaffolds facilitate lamellar bone formation and collagen alignment, reinforcing their value in regenerative medicine [95].

Table 2 highlights the critical physicochemical properties of PLGA-based implants, such as porosity, molecular weight, degradation profiles, and release kinetics, which are essential for tailored drug delivery and therapeutic efficacy. These properties are meticulously engineered, linking structural and functional components to achieve optimized therapeutic outcomes. For example, controlled porosity and molecular weight influence degradation rates and drug release patterns, while hydrophilicity and polymer ratios affect swelling and drug mobility. Stability under physiological conditions ensures long-term effectiveness, while structural integrity supports applications in bone repair and tissue scaffolding.

## 4. Fabrication Techniques for Tailored PLGA Implants

The versatility of PLGA-based implants stems from a broad array of fabrication techniques that allow precise control over drug release, mechanical strength, and biological interactions. These methods integrate structural, therapeutic, and functional elements, enabling custom-tailored systems for specific clinical applications.

### 4.1. Solvent-Based Fabrication Techniques

Solvent-based methods are extensively employed in PLGA implant design. Phase separation using solvents such as dimethyl sulfoxide (DMSO) enables precise drug incorporation and controlled solidification [61,82]. Coacervation with dichloromethane improves drug encapsulation efficiency, while solvent exchange systems with N-methyl-2-pyrrolidone (NMP) and glycofurol facilitate sustained in situ formation [23,53,82]. A novel reversed-phase separation/coacervation approach produced flexible ciprofloxacin-loaded PLGA implants capable of 65-day antibiotic release [82]. Microfabrication technologies like UV-LIGA have been utilized to engineer cisplatin implants with micro-chamber structures for precise release modulation [113].

### 4.2. Hot Melt Extrusion (HME) for Controlled Release

**Figure 2:**
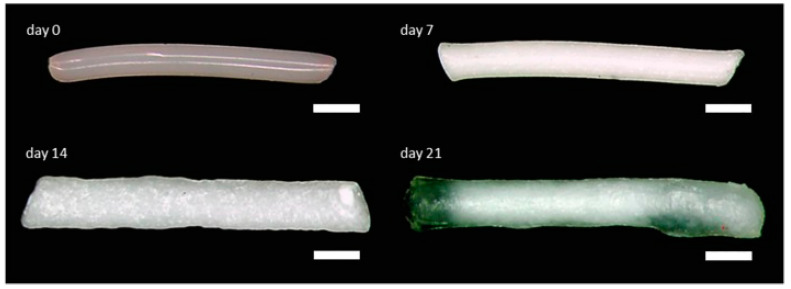
The morphology of a biodegradable dexamethasone-loaded PLGA implant prepared by hot melt extrusion, shown at day 0 and after incubation in PBS at day 7, day 14, and day 21, that provided controlled 80% of intracochlear drug release for up to 3 weeks. Scale bar indicates 500 µm. Adopted with permission [33].

Hot melt extrusion (HME) offers a solvent-free fabrication route, promoting uniform drug distribution and mechanical stability. Plasticizers improve porosity and drug release rates, as demonstrated in implants loaded with ibuprofen [4], dexamethasone [22,68], and BSA [85]. Mini-scale HME also supports bioactive protein encapsulation [104]. Figure 2 shows the morphological evolution of a dexamethasone-loaded PLGA implant over 21 days in PBS [33]. HME has also proven effective for creating long-acting drug delivery systems, including ovalbumin-based proteins, monoclonal antibodies [42], and gentamicin sulfate implants for osteomyelitis, with encapsulation efficiencies of 85–115% and sustained profiles comparable to PMMA-based Septopal^®^ [70].

### 4.3. In Situ Forming and Injectable Implants

In situ forming implants (ISFIs) use solvent exchange or phase inversion to solidify PLGA in vivo, enabling minimally invasive and sustained drug delivery. Leuprolide acetate ISFIs reduce burst release while maintaining prolonged release [64,88,101]. Solvents like NMP and DMSO control solidification and long-term kinetics, particularly in oncology and psychiatric treatments [15,55,114]. Injectable formulations form gel matrices upon contact with body fluids, offering tunable release profiles [115]. For instance, ISFIs incorporating rosuvastatin and copper–selenium nanoparticles achieved combined antimicrobial and anticancer activity for breast cancer treatment [43].

### 4.4. Advanced 3D Printing Techniques

**Figure 3:**
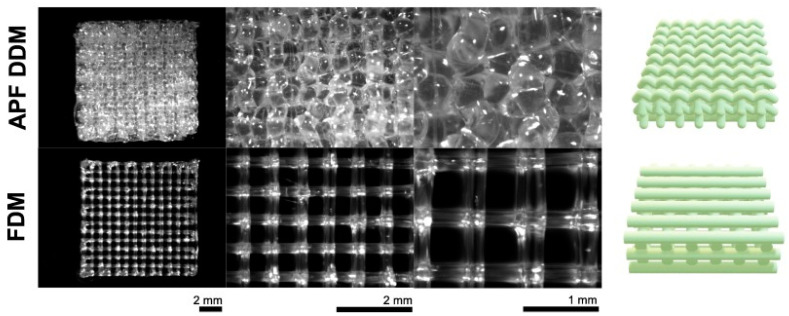
Optical macroscopy pictures of ibuprofen-loaded PLGA implants (meshes) prepared with the use of Droplet Deposition Modeling (APF DDM) or Fused Deposition Modeling (FDM), before being exposed to the release medium. On the right-hand side are illustrations of the real structures of the mesh-shaped implants. Adopted with permission [59].

Three-dimensional printing enables precise control over implant geometry, porosity, and drug loading. Technologies such as fused deposition modeling (FDM) and Arburg Plastic Freeforming (APF) are used to create ibuprofen-loaded PLGA meshes with controlled release characteristics [17,59]. Figure 3 shows optical macroscopy images of these printed meshes [59]. Comparative studies show that FDM yields more porous structures with faster release, whereas APF enables extended profiles. Infill density is a critical parameter affecting release kinetics, particularly in bone regeneration scaffolds [106].

### 4.5. Microspheres, Nanoparticles, and Scaffold Fabrication

Microspheres and nanoparticles allow precise drug encapsulation and controlled release. Oil/water emulsion techniques support the loading of proteins and growth factors such as cytochrome C and bFGF [36,83,116]. Microfluidic emulsification improves monodispersity and release control [107], while spray drying and freeze milling ensure stability and mechanical integrity in wafers and particles [10,86]. Hybrid PLGA particles have been engineered for enhanced therapeutic effects, including pH-responsive curcumin nanoparticles via nanoprecipitation and lipid–PLGA systems for antipsychotic delivery [117]. Melt-grafted PLGA with hydroxyapatite (HA) or β-tricalcium phosphate (β-TCP) improves mechanical properties and regenerative potential [49,99]. Scaffolds and plugs are fabricated through direct printing or extrusion methods for applications in bone regeneration and annular defect repair [95,118]. PLGA/fibrin scaffolds support nerve ingrowth, particularly in disk degeneration therapies [52].

### 4.6. Surface Engineering and Hybrid Systems

Surface coating and layering techniques optimize implant performance through improved biocompatibility and drug delivery. Electrospraying and dip-coating methods are used to apply uniform PLGA layers on titanium and chitosan substrates for targeted antibiotic or growth factor release [35,90,102]. Ultrasonic spray coating allows testosterone–alendronate integration for orthopedic use [94]. Microsphere attachment at room temperature enhances antibacterial efficacy on titanium surfaces without compromising osteoblast function [119]. Microneedle-based implants fabricated via casting-mold techniques enable self-administered levonorgestrel (LNG) release with strong mechanical properties [75,109].

### 4.7. Real-Time Monitoring and Structural Optimization

Innovative designs and imaging tools enable real-time feedback and structural optimization of PLGA implants. Honeycomb-like microstructures fabricated using microlithography support linear drug release patterns [120]. MRI and EPR spectroscopy allow non-invasive tracking of degradation and drug distribution, facilitating the development of more predictable delivery systems [93].

Table 3 details the processing methods and manufacturing techniques used for PLGA-based implants, focusing on techniques like solvent evaporation, microfluidics, and hot melt extrusion, and their impacts on drug encapsulation and implant properties. Processing methods play a pivotal role in defining the efficacy of PLGA-based implants. Solvent evaporation ensures uniform particle size, while microfluidics enhances drug loading efficiency. Techniques like hot melt extrusion allow for high-temperature processing, supporting controlled release. Innovations like 3D printing enable implants tailored to individual anatomical needs. These methods also optimize implant integrity, shape, and biocompatibility, demonstrating the industry’s emphasis on precision engineering.

## 5. Testing and Validation of PLGA Implants

Comprehensive testing and validation are critical for ensuring the safety, efficacy, and clinical applicability of PLGA-based implants. Evaluations encompass physicochemical characterization, drug release behavior, biocompatibility, degradation kinetics, and therapeutic performance across diverse biomedical contexts.

### 5.1. Drug Release and Kinetics

Drug release assessments are foundational in validating implant performance. In vitro and in vivo studies characterize initial burst, sustained release, and long-term stability [1,40,76,96]. Pharmacokinetic evaluations, including plasma concentration analysis and in vitro-in vivo correlation (IVIVC), confirm the reliability of drug delivery [20,134,137]. Additionally, swelling and degradation studies inform drug stability and therapeutic window optimization [22,54,128].

### 5.2. Material Properties and Fabrication

Material characterization techniques are essential for assessing implant structure–function relationships. Thermal and mechanical properties are analyzed using differential scanning calorimetry (DSC), X-ray diffraction (XRD), and tensile testing [16,30,82]. Surface morphology and porosity—key to coating stability and release kinetics—are examined via scanning electron microscopy (SEM) and gravimetric analysis [21,29,57]. For precision manufacturing, 3D printing and microfabrication outputs are validated for geometric accuracy and release control [42,106,120]. Nanoparticles and microspheres are evaluated for particle size, zeta potential, and encapsulation efficiency to ensure consistency and bioavailability [3,108,138].

### 5.3. Degradation and Stability Studies

PLGA degradation studies evaluate implant longevity and functional stability. Polymer composition, additives, and environmental factors influence degradation rates, assessed through mass loss, pH shifts, and erosion analysis [95,139,140]. Both real-time and accelerated degradation protocols provide insights into behavior under physiological and stress conditions [111,141]. In vivo subcutaneous and intrathecal models further confirm structural integrity and degradation in biological systems [142,143].

### 5.4. Biocompatibility and Toxicity

Biocompatibility is validated via histological analysis and cytotoxicity assays to ensure minimal inflammatory response and optimal tissue integration [28,144,145]. Functional enhancements, including anti-inflammatory coatings and bioactive agents, contribute to reduced tissue reactivity. Long-term toxicity studies assess systemic and localized safety in ocular and systemic therapies [112,123,137]. In regenerative contexts, histomorphometric analyses evaluate tissue integration, fibrosis suppression, and adipose tissue formation [141,146].

### 5.5. Application-Specific Evaluations and Therapeutic Performance

**Figure 4:**
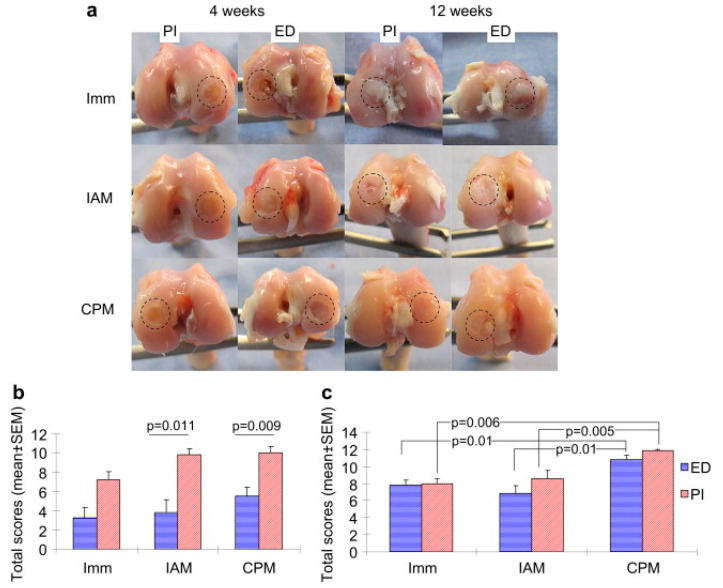
The gross appearances of osteochondral regeneration in rabbit in three treatments: continuous passive motion (CPM), immobilization (Imm) and intermittent active motion (IAM) with PLGA implant (PI) and without PLGA implant as empty defect (ED) at weeks 4 and 12 postoperatively. The circle on the diagram shows the area of focus for osteochondral regeneration for each treatment. (**a**) Shows the quantitative scores of gross appearances at 4 weeks. (**b**) Shows the quantitative scores of gross appearances at 12 weeks. (**c**) Shows the quantitative scores of gross appearances after surgery. Adopted with permission [148].

PLGA implants are rigorously tested for efficacy in their target medical applications. In bone regeneration, micro-computed tomography (micro-CT) and histological methods assess osteogenic differentiation, bone–implant contact, and peri-implant growth [36,48,147]. Cartilage repair studies focus on glycosaminoglycan (GAG) content, collagen fiber alignment, and osteochondral integration [148,149]. Figure 4 illustrates the impact of continuous passive motion combined with acellular PLGA implants on osteochondral healing in rabbits [148].

In neural applications, disk height index, vascular proliferation, and functional recovery are evaluated through spinal repair models [52,118,150]. Antimicrobial efficacy is tested using infection models involving nanosilver or vancomycin-loaded systems [29,37,45]. Functional assessments such as tail-flick, paw-withdrawal, and locomotor recovery quantify therapeutic impact in spinal and intrathecal delivery [143,150,151]. Antiangiogenic activity is analyzed through CAM assays and HUVEC migration, particularly in ophthalmology and oncology [38,39]. Clinical outcomes, complication rates, and overall effectiveness are evaluated in patient trials [67,152,153].

### 5.6. Imaging and Monitoring Techniques

Advanced imaging supports real-time analysis of implant behavior. MRI, micro-CT, and X-ray imaging provide structural and bioactivity data critical for tissue integration studies [33,148,152]. Molecular characterization tools such as FTIR and HPLC-UV elucidate drug–polymer interactions and degradation profiles. Immunofluorescence imaging captures cellular responses, aiding the validation of therapeutic efficacy and biocompatibility [39,103,125].

### 5.7. Statistical and Computational Approaches

Computational modeling and statistical analysis facilitate implant design and performance validation. Finite element modeling simulates drug diffusion, polymer degradation, and mechanical behavior. Statistical methods, including Box–Behnken design and ANOVA, identify optimal formulation parameters and ensure reproducibility [55,84,136]. These approaches support precision engineering and predictive evaluation of PLGA systems.

Table 4 lists the diverse testing and evaluation methodologies employed for PLGA-based implants, including in vitro, in vivo, and imaging-based tests to validate their release profiles, biocompatibility, and therapeutic efficacy. These methods evaluate how polymer matrices, additives, and therapeutic agents work together to optimize implant performance. In vitro studies assess encapsulation efficiency, release kinetics, and degradation rates, while in vivo analyses confirm biocompatibility and therapeutic outcomes. Advanced imaging techniques, such as MRI and fluorescence microscopy, provide real-time insights into implant behavior. Biomechanical and histological tests ensure implant integration and tissue compatibility. This comprehensive validation guarantees that PLGA implants meet clinical and regulatory standards, ensuring their safety and efficacy for patient use.

## 6. Tailored Therapeutic Effects of PLGA Implants

PLGA implants can be precisely engineered to respond to a range of physical and biological stimuli, enabling controlled drug release, site-specific action, and improved therapeutic outcomes. This section presents studies that highlight how PLGA implants are tuned to exogenous and endogenous triggers to optimize clinical performance.

### 6.1. Exogenous Stimuli

External stimuli such as ultrasound, mechanical forces, temperature, magnetic fields, and light have been used to enhance drug release dynamics, guide tissue regeneration, and modulate implant degradation. These approaches offer non-invasive and controllable strategies for improving treatment efficacy. However, their clinical feasibility depends on factors such as safety, regulatory clearance, and device compatibility.

Ultrasound: Ultrasound enhances PLGA degradation and swelling, enabling externally modulated drug release. Ultrasound-assisted imaging has been used to monitor phase inversion, correlating it with drug release kinetics [5]. In fluorescein-loaded PLGA systems, ultrasound improved drug penetration by 1.7- to 5.6-fold in soft tissue-mimicking environments, but not in PBS, indicating its effectiveness in biological matrices [158]. When combined with high-intensity focused ultrasound (HIFU), PLGA implants releasing doxorubicin improved tumor penetration, apoptosis, and shrinkage over HIFU treatment alone [159]. Notably, these applications utilize clinically established modalities, and ultrasound’s non-invasive nature and real-time imaging compatibility make it one of the most translatable external triggers.

Magnetic Fields: Magnetic field-responsive PLGA systems enable localized therapy with high precision. A PLGA(Ag-Fe_3_O_4_)-coated dental implant promoted osteogenesis and antibacterial activity under magnetic stimulation [44]. Injectable PLGA/Fe_3_O_4_ implants carrying cisplatin generated localized hyperthermia under alternating magnetic fields (AMF), leading to tumor necrosis and immune activation [72]. Similarly, phase-transitional PLGA-Fe implants responded to AMF by triggering doxorubicin release and tumor cell death, with no leakage and visibility under CT and ultrasound, meeting essential safety and monitoring requirements [74]. These systems demonstrate clinical potential through their integration with imaging tools and effective retention at target sites [159].

Light-Responsiveness: Light-activated PLGA systems allow spatiotemporally controlled drug release. Methacrylated alginate (MA)-PLGA ISFIs were photocrosslinked to achieve sustained intravitreal peptide delivery, with excellent biocompatibility and suitability for ophthalmic applications [108]. Such technologies are promising for minimally invasive and localized drug delivery, particularly where external light application is already routine, such as in ophthalmology and dermatology.

Mechanical Forces: Mechanical properties affect drug diffusion and implant behavior. Larger implant diameters slow drug diffusion by enhancing mechanical stability [8]. Continuous passive motion (CPM) combined with acellular PLGA scaffolds enhanced osteochondral regeneration in rabbits, with improved collagen expression and bone formation [148]. A PLGA–graphene scaffold increased bone volume by 50% in rats [129], while a bilayered PVA/PLGA shuttle supported deeper neural probe insertion for chronic electrophysiology [160]. Shape design, such as the “basket-in-tube” structure, stabilized release kinetics by minimizing implant movement [84]. Electrospun PLGA nanofibers co-loaded with fusidic acid and rifampicin prevented MRSA and S. epidermidis colonization on titanium implants [128].

Temperature Modulation: Temperature affects polymer porosity and drug diffusion. While heat exposure altered crystalline content in ibuprofen-loaded PLGA, it had a minimal impact on overall release [4]. Higher temperatures increased porosity and reduced burst release, while lower temperatures caused denser matrices and faster initial release [9]. Temperature also influenced microclimate pH, impacting hydrophilic drug stability during degradation [18].

In summary, while all stimuli-responsive systems offer promising control over drug release, ultrasound and magnetic field-based systems currently show the highest clinical feasibility. This is due to their non-invasive nature, established use in clinical imaging and therapy, and compatibility with real-time monitoring tools. Importantly, these modalities often leverage FDA-cleared devices, potentially reducing regulatory barriers. Their integration with implantable drug delivery systems has demonstrated both preclinical efficacy and controlled, localized drug activation. Continued progress in device integration, standardized protocols, and regulatory alignment will be essential to fully realize their clinical potential.

### 6.2. Endogenous Stimuli

PLGA implants are also responsive to biological stimuli such as enzymes, pH, inflammatory signals, and disease-specific environments. These factors influence degradation, drug release behavior, and host interaction, allowing for context-specific therapeutic adaptation.

Enzyme Sensitivity: Endogenous enzymes modulate PLGA degradation and drug release kinetics. Rilpivirine- and paclitaxel-loaded implants showed enzyme-mediated alterations in release profiles [2,6,13]. Lysozyme accelerated degradation in PLGA-PEG block copolymers, though co-encapsulated MgCO_3_ buffered acidic byproducts and preserved protein integrity [24]. BMP-2 release from layer-by-layer coated PLGA implants was enzyme-regulated, enhancing osteoblast activity [97].

pH Sensitivity: PLGA degradation produces acidic byproducts that affect stability. Dexamethasone-loaded PLGA systems exhibited pH-dependent release governed by glycolic acid content and end-group chemistry [68]. Acidic environments caused protein aggregation in PLGA matrices, countered by shellac incorporation for stabilization [11]. Swelling-induced osmotic pressure led to up to 1700% volume expansion, delaying release due to hydrophobic resistance to water infiltration [22]. PLGA/PLA nanoparticles modified with pH-sensitive surfactants improved antibacterial activity and corrosion resistance [71].

Disease Microenvironment Sensitivity: Implant behavior varies with disease context. In spinal cord injury models, scaffold stiffness influenced inflammation and neuroprotection, with softer scaffolds promoting regeneration [157]. In stroke models, VEGF-loaded PLGA microparticles with hNSCs enhanced vascularization and neural growth, though some hypervascularization was noted [34]. In Alzheimer’s disease, VEGF-loaded nanospheres increased hippocampal neurogenesis [79]. In cancer, doxorubicin-loaded implants improved local tumor suppression in osteosarcoma [161], and 5-FU-loaded PLGA systems sustained peritoneal concentrations in colon cancer while minimizing systemic toxicity [162].

Inflammatory Response and Immune Modulation: PLGA interactions with immune cells influence biocompatibility. SIS scaffolds elicited less inflammation than PLGA, while SIS/PLGA hybrids showed intermediate responses [50]. PLGA/PVA hydrogels with dexamethasone extended anti-inflammatory release for up to 95 days [146]. Predegraded dexamethasone microspheres reduced foreign body reactions when integrated into sutures [69].

Bone Regeneration and Osseointegration in Disease Contexts: PLGA implants support bone healing in compromised environments. bFGF-loaded microspheres improved osseointegration in diabetic rats [36], and exendin-4-loaded chitosan–PLGA microspheres promoted osteogenesis in diabetic models [48]. Zoledronate-loaded PLGA microcapsules reduced bone loss and inflammation in periodontitis by modulating cytokine expression [163]. A PLGA-coated implant releasing testosterone and alendronate enhanced mineralization and bone–implant contact [94].

Ocular and Neurological Applications: PLGA systems provide sustained release for chronic ocular diseases. Tacrolimus-loaded PLGA implants delivered the drug over six weeks with no ocular toxicity [137]. Lupeol-loaded PLGA systems suppressed endothelial proliferation and neovascularization in macular degeneration [38]. For glioblastoma, paclitaxel-loaded implants released the drug over 42 days, reducing tumor volume by 30-fold with 5 mm diffusion from the implant site [131].

## 7. Applications and Benefits of PLGA-Based Implants

PLGA-based implants are pivotal in modern biomedicine, offering precision-controlled drug release, support for tissue regeneration, and effective infection management. Their tunable design—enabled by structural modifications, functional additives, and drug–polymer integration—has expanded their clinical utility across diverse therapeutic domains.

### 7.1. Sustained and Controlled Drug Release

PLGA implants enable prolonged, programmable drug release, reducing dosing frequency and improving patient adherence. Customizable release profiles incorporating burst, diffusion, and erosion phases support precise therapeutic control [1,2,15,82,115]. For instance, rilpivirine implants extend HIV-1 therapy to 42 days [2], while entecavir implants maintain therapeutic levels for hepatitis B with 77.84% drug entrapment efficiency [1]. Tailoring molecular weight and additives like PEG and poloxamer allows optimization of drug kinetics across applications [3,10,12,164].

### 7.2. Localized and Minimally Invasive Drug Delivery

PLGA implants enhance site-specific delivery while minimizing systemic side effects. Ibuprofen-loaded implants offer localized analgesia [4], and tamsulosin implants release the drug over 10 days with reduced systemic exposure [61]. In oncology, PLGA-based systems deliver paclitaxel, carboplatin, and doxorubicin directly to tumor sites, improving efficacy and reducing toxicity [6,47,165]. VEGF-loaded PLGA implants support neurogenesis in stroke and Alzheimer’s models [34,79].

Innovative platforms like in situ forming implants (ISFIs) bypass gastric degradation and improve insulin bioavailability in diabetes therapy [166]. Microneedle-based PLGA systems enable long-term contraceptive delivery through minimally invasive methods [109]. These delivery routes have also been applied in glioblastoma and colon cancer treatments [6,122,165].

### 7.3. Mitigation of Burst Release and Drug Stability

**Figure 5:**
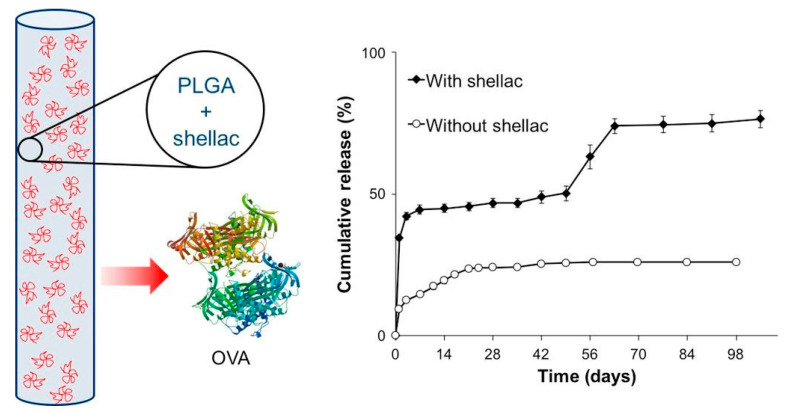
PLGA-based implants for the acid-labile model protein ovalbumin with the use of shellac polymer as a protective excipient. This implant demonstrated a triphasic release profile providing a slow diffusion phase over 7 weeks and an erosion-controlled dissolution phase for 3 weeks. Adopted with permission [11].

PLGA formulations are engineered to minimize initial burst release and enhance the stability of labile drugs. Excipients like albumin–oleic acid conjugates and beta-cyclodextrin improve release profiles and reduce toxicity [12,62,167]. PLGA also stabilizes sensitive biologics, including human growth hormone (hGH) and insulin, preserving therapeutic efficacy [166,168]. Among various techniques, end-group modification—such as using ester-terminated PLGA—has shown significant promise by reducing hydrophilicity and early water uptake, thereby lowering burst release in clinically relevant formulations [2]. Phase inversion control, through manipulation of temperature and solvent systems, also minimizes burst by forming denser matrix structures during implant formation [9,56,88]. Additionally, PEG-based copolymer blends like PLGA-PEG-PLGA reduce burst release and align well with pharmacokinetic profiles of marketed products, supporting their clinical translatability [127]. Figure 5 shows a schematic of a PLGA-based implant incorporating shellac as a protective excipient for encapsulating the model protein ovalbumin (OVA), alongside a graph illustrating the cumulative release profile over 98 days. The triphasic release behavior, enabled by the integration of shellac as a material component, demonstrates how such modifications optimize drug delivery. Implants without shellac, in contrast, show significantly reduced protein release over the same period [11].

### 7.4. Advances in Regenerative Medicine

PLGA scaffolds are widely applied in bone and dental tissue engineering. The integration of β-TCP and hydroxyapatite enhances bone regeneration and osseointegration, supporting femoral defect and dental repair [65,99]. Antibacterial agents such as nanosilver and Ag-Fe_3_O_4_ nanoparticles improve implant safety and promote osteoblastic activity [44,45]. In neural applications, flexible PLGA-coated devices reduce glial scarring and inflammation, promoting neural interface integration [89]. Growth factor-loaded scaffolds containing BDNF or VEGF accelerate regeneration in spinal cord injury models, demonstrating promise in neurorehabilitation [151,157].

### 7.5. Specialized Applications

PLGA systems address a range of specialized medical needs. PEG-modified PLGA implants enhance radiosensitization in hypoxic tumors, optimizing therapeutic outcomes in cancer [3]. Antibiotic-loaded PLGA implants, including Eudragit-modified systems, extend treatment efficacy against Pseudomonas aeruginosa for up to 12 days [87]. The precise delivery of chemotherapeutics and growth factors positions PLGA as a leading material for treating gliomas, breast cancer, and neurovascular disorders [6,47,165].

### 7.6. Enhancing Biocompatibility and Safety

Biocompatibility is enhanced through surface engineering and additive incorporation. PEG-modified PLGA and SIS scaffolds mitigate the acidic byproducts of degradation, reducing inflammatory responses [24,50,68]. These strategies are especially relevant in neural and ocular applications, where inflammation is a major concern. PLGA-PEG copolymers buffer local acidity and support sustained protein release with reduced immune activation [24], while SIS blending significantly attenuates host tissue responses compared to PLGA alone [50]. Surface coatings, such as PLGA-Parylene C, and shape-optimized designs improve mechanical integrity while refining drug release characteristics [84,103,119]. Additionally, attaching PLGA microspheres to implant surfaces enables localized drug delivery with antibacterial effects and promotes cell adhesion, further supporting long-term biocompatibility [119].

Table 5 emphasizes the proven benefits of PLGA-based implants, from controlled drug release and biocompatibility to enhanced patient compliance, highlighting their contributions to diverse therapeutic and diagnostic advancements. Their versatility arises from the ability to tailor structural and functional components to meet specific clinical needs. PLGA-based implants offer unparalleled advantages, including extended drug release, reduced dosing frequency, and localized treatment with minimal systemic effects. Their biocompatibility ensures safe degradation into metabolizable components. Through customized designs, such as modifying shape, surface characteristics, and release profiles, these implants are optimized for precision medicine approaches, addressing chronic and complex diseases across oncology, neurology, and orthopedics. Customizable properties, like shape and release profiles, enable precision medicine approaches. Moreover, their integration of diagnostic and therapeutic capabilities (theranostics) demonstrates the cutting-edge potential of PLGA implants. By combining therapeutic effects with diagnostic functionality, PLGA implants represent a cutting-edge solution for modern healthcare challenges, significantly improving patient outcomes and quality of life.

Table 6 explores the diverse application areas of PLGA-based implants across medical fields. It categorizes implants based on therapeutic domains like oncology, endocrinology, neurology, and orthopedics, providing insights into how these implants support targeted treatments and long-term drug delivery. PLGA-based implants are versatile, addressing chronic conditions requiring sustained delivery systems. Oncology benefits from localized therapies that minimize systemic side effects, while endocrinology employs implants for hormonal regulation. Neurology applications focus on neural repair and inflammation control, and orthopedics utilizes implants for tissue regeneration and infection prevention. The ability to customize PLGA systems through the incorporation of functional materials ensures their adaptability to unique medical challenges, while their biodegradability ensures minimal residual toxicity, reinforcing their role as trusted systems for systemic and local treatments.

## 8. Customization of Implants for Individual Patients

Customized PLGA implants play a critical role in advancing personalized medicine by addressing patient-specific variables such as age, gender, disease condition, tissue type, and defect size. Recent advances in fabrication methods, computational modeling, imaging techniques, and smart materials have enabled the development of biomimetic implants tailored for individualized treatment.

### 8.1. Personalized Medicine Factors: Age, Gender, Health, and Defect Characteristics

Bone Regeneration and Healing Response: Bone healing capacity varies based on factors such as age, metabolic condition, and tissue density. Insulin-loaded PLGA microspheres enhanced peri-implant bone regeneration in diabetic conditions, demonstrating the value of customized drug release in compromised healing environments [7]. β-TCP/PLGA root analogs showed successful bone-like tissue formation after three months, supporting the application of personalized dental implants [65]. In diabetic models, PLGA-gold nanoparticle (AuNP) systems delivering antagomiR204 improved osseointegration, addressing disease-specific healing deficiencies through gene-modulated therapy [46]. Exendin-4-loaded chitosan–PLGA microspheres also enhanced osteogenic differentiation, further confirming the utility of targeted drug release for metabolic bone disorders [48].

Optimized Drug Delivery for Individual Needs: Customized drug release kinetics can significantly impact therapeutic efficacy. A Box–Behnken design optimized an injectable risperidone PLGA implant, minimizing burst release and achieving 51.08% drug release over 40 days—an alternative to conventional microsphere-based formulations for schizophrenia [55]. Similarly, PLGA-loaded mitomycin C (MMC) implants supported glaucoma surgery by sustaining intraocular pressure reduction [175]. Structural variations in PLGA-based dexamethasone intravitreal implants influenced release behavior, underlining the need for physiologically relevant test models [140].

### 8.2. Biomimetic Design: Scaffold Rigidity, Pore Size, and Implant Shape

Tissue-Specific Rigidity Optimization: PLGA scaffold stiffness plays a pivotal role in tissue-specific regeneration. In spinal cord injury (SCI) repair, soft scaffolds promoted neuroprotection, while stiffer versions induced mesodermal differentiation, highlighting the need for application-specific rigidity [157]. In cartilage engineering, MSC-seeded PLGA scaffolds exhibited time-dependent regeneration, reinforcing the importance of patient- and defect-specific implant design [173]. Flexible PLGA scaffolds seeded with chondrocytes supported meniscal healing under both static and dynamic conditions in a mouse model [176].

Optimizing Pore Size and Surface Characteristics: Pore size and surface architecture are pivotal in guiding tissue integration and regeneration. PLGA scaffolds with pores in the range of 300–500 µm have been shown to optimally support lamellar bone formation and promote collagen and hydroxyapatite deposition, outperforming both smaller and larger pore sizes [95]. Complementarily, surface-engineered implants—such as PLGA films deposited on TiO_2_ nanotube arrays—enable sustained release of osteogenic factors like rhBMP-2. A 250 nm thick PLGA layer demonstrated controlled release over four weeks while significantly enhancing osteoblast adhesion, proliferation, and differentiation, underscoring the potential of nanoscale modifications for targeted bone healing [35].

### 8.3. Biomechanical and Environmental Influences on Custom Regeneration

Exercise-Based Enhancement of Tissue Healing: Mechanical stimulation can significantly enhance implant performance. Acellular PLGA scaffolds combined with treadmill activity improved cartilage regeneration in minipigs [149]. Similarly, early treadmill exercise in rabbits accelerated cartilage repair, reduced inflammation, and enhanced trabecular integration when used with PLGA scaffolds [78].

Customized Pain Management: For postoperative applications, PLGA implants delivering racemic bupivacaine or Novabupi^®^ provided 30-day pain relief. Variations in polymer composition modulated release kinetics, validating the importance of personalized implant formulations for analgesia [139].

### 8.4. Advanced Technologies for Personalized PLGA Implants

Computational Modeling for Implant Design: Finite element modeling has been used to simulate drug transport in PLGA nanofiber systems, allowing for precise prediction of release profiles [136]. A systematic review introduced a “12 Factor System” to optimize in situ forming PLGA implants, offering a standardized framework for personalized drug delivery customization [167].

Imaging Technologies for Implant Optimization: Imaging advances facilitate non-invasive, real-time monitoring of implant behavior. MRI and EPR spectroscopy enabled the tracking of degradation and drug release in vivo [93]. Ultrasound imaging further aided in monitoring swelling and degradation profiles in injectable systems [5]. Fluorescence imaging helped assess microclimate pH inside PLGA implants, improving understanding of in vivo drug stability [18].

### 8.5. Integration of Functionalized Nanoparticles for Patient-Specific Needs

Nanoparticles for Disease-Specific Applications: Functionalized nanoparticles enhance biocompatibility and targeted release for individual patients. Light-responsive PLGA hybrid nanoparticles enabled controlled peptide release in intravitreal therapy, improving retention and compliance in ocular disease management [108]. For cancer therapy, DOX-loaded PLGA implants were adapted for intrahepatic administration, achieving high local drug levels while minimizing systemic exposure [26]. Cannabidiol (CBD)-loaded PLGA implants provided sustained release for one month and exhibited antiangiogenic effects in tumor models, supporting patient-specific cannabinoid-based therapies [178].

## 9. Challenges in the Development of PLGA Implants

Despite significant advancements, PLGA-based implants still face critical challenges that limit their clinical adoption and long-term effectiveness. These include inconsistencies in drug release, inflammatory responses, structural and mechanical limitations, and difficulties in achieving long-term therapeutic efficacy. Understanding these barriers—along with insights from unsuccessful or limited studies—can inform future innovation in PLGA implant technology.

### 9.1. Challenges in Drug Release Consistency and Predictability

Burst Release and Protein Aggregation: One of the most prominent issues in PLGA systems is burst release, particularly problematic in sensitive applications like ocular or cancer therapy [2,15,16,54,55,57,62,101,164]. Incomplete protein release is also common due to aggregation in acidic microenvironments. Ovalbumin (OVA)-loaded implants demonstrated limited diffusion caused by polymer interactions, while the incorporation of shellac improved release efficiency by neutralizing acidic degradation products [11]. Interactions between proteins and the PLGA matrix further contributed to incomplete release, emphasizing the need for formulation strategies to mitigate these effects and promote bioavailability [85].

Unexpected Burst and Lag Times in ISFIs: Controlling release kinetics remains challenging in in situ forming implants (ISFIs). Dexamethasone-loaded PLGA microspheres initially exhibited rapid release due to predegradation, followed by reduced delivery after two weeks, complicating sustained release strategies [40]. Similarly, PLGA-based ISFIs with medium molecular weight exhibited higher burst release due to increased porosity [101]. Solvent interactions and polymer composition significantly affect release patterns, limiting reproducibility [53].

IVIVC Challenges: Establishing predictive in vitro–in vivo models remain difficult. In risperidone ISFIs, higher molecular weight PLGA and lactide content extended drug release, but IVIVC failed due to unpredictable phase separation [20]. In tamsulosin-loaded PLGA implants, the inclusion of Tween and Span affected kinetics, but inconsistent release profiles hindered predictability, underlining the need for improved modeling tools [61].

### 9.2. Manufacturing Complexity and Cost Barriers

Advanced techniques like hot melt extrusion, electrospraying, and 3D printing improve precision but require specialized equipment and expertise, raising production costs and limiting scalability [4,11,17,59,72,102,108,111,128]. Efforts to replace solvents like NMP in ISFIs with PEG 400 or triethyl citrate improved syringeability, but drugs like ibuprofen still exhibited burst release, while chlorhexidine showed lag phases, revealing ongoing formulation challenges [58].

### 9.3. Limitations in Bone Regeneration and Tissue Engineering

Suboptimal Bone Integration Outcomes: While widely applied in orthopedic contexts, PLGA sometimes fails to deliver expected outcomes. Lithium-incorporated PLGA implants aimed at Wnt signaling modulation did not enhance early bone growth [14]. Comparisons of nano- and microsized β-TCP in PLGA revealed limited osteointegration and lower mechanical strength versus titanium alloy implants [99]. Additionally, β-TCP/PLGA spacers used in knee osteoarthritis demonstrated some clinical benefits but were limited by degradation inconsistencies and mechanical instability under load [67].

Short-Term Regenerative Effects of Cell-Seeded Scaffolds: Mesenchymal stromal cell (MSC)-seeded PLGA scaffolds showed declining effectiveness in cartilage repair over 12 months [173]. In chondrocyte-seeded PLGA scaffolds, fibrocartilaginous repair occurred under static and dynamic conditions, but the results highlighted the critical influence of biomechanical stimulation [176]. Similarly, preadipocyte-seeded PLGA scaffolds initially supported adipose tissue formation, but by 5–12 months, both implant and tissue had degraded, indicating poor long-term viability [141].

### 9.4. Inflammatory Reactions and Biocompatibility Issues

Persistent Inflammatory Responses: PLGA scaffolds can trigger notable inflammatory reactions. Studies comparing PLGA to porcine small intestinal submucosa (SIS) showed milder inflammation in SIS scaffolds, suggesting a need for surface modifications or material hybrids to improve host response [50]. In intracerebral applications, carboplatin-loaded PLGA microspheres induced localized inflammation [165], while larger PLGA-based neural microelectrodes caused tissue adhesion, reinforcing the importance of structural refinements [89].

Excessive Vascularization and Unintended Outcomes: In some cases, PLGA-based implants cause unintended biological effects. VEGF-releasing PLGA microparticles used in stroke models increased vascularization but also led to hypervascularization in certain areas, requiring careful dose regulation [34]. In another study, PLGA/fibrin gel scaffolds implanted in intervertebral disks resulted in increased nerve fiber ingrowth, potentially contributing to pain rather than healing [52].

### 9.5. Structural and Mechanical Limitations of PLGA Implants

Mechanical Weakness in Load-Bearing Applications: Mechanical insufficiency remains a limitation in orthopedic use. While PLGA microparticles in calcium phosphate (CaP) cement improved bone ingrowth, mechanical strength was inadequate for load-bearing roles [147]. Attempts to enhance PLGA surface properties via CO_2_ laser modification improved degradation behavior, but high exposure levels caused excessive degradation and weakened tensile strength, restricting use in high-stress environments [111].

## 10. Opportunities and Future Directions in PLGA Implant Research

Despite current challenges, ongoing advancements in polymer science, fabrication techniques, and functional design are rapidly expanding the future clinical potential of PLGA-based implants. Innovations such as polymer blending, multilayered architectures, and functional coatings are enabling better control over drug release, reducing burst effects, and improving therapeutic stability and long-term efficacy [19,57,88,121,164,167].

Looking ahead, a number of strategic opportunities are emerging. There is growing interest in engineering implants that respond to local microenvironments—such as pH shifts or enzymatic activity—or to exogenous stimuli like ultrasound and magnetic fields, enabling on-demand therapeutic modulation. Functional integration with biosensors or imaging agents could lead to theranostic platforms, paving the way for intelligent and responsive implants. In parallel, materials science is converging with biomedical engineering to design next-generation PLGA composites tailored for specialized applications in the brain, inner ear, and ocular compartments.

Scalability is being addressed through cost-effective manufacturing techniques, including advanced 3D printing and microfluidic platforms, which are making production more accessible and customizable [4,13,17,59,72]. However, scaling these technologies for clinical use presents challenges. In 3D printing, differences in deposition methods such as Droplet Deposition Modeling (DDM) and Fused Deposition Modeling (FDM) significantly affect implant porosity and drug release, even with identical designs [17]. High filling densities limit drug diffusion, while low-density meshes support faster release [59]. Thermal processes like hot melt extrusion can also alter polymer and drug stability, requiring tight process control [4]. Microfluidics offers precise particle fabrication but faces limitations in throughput and scalability [13]. Despite these barriers, such methods remain promising for personalized PLGA-based therapies with continued optimization.

Enhancing biocompatibility remains a core objective. Future efforts will increasingly rely on integrating biocompatible additives such as chitosan, hydroxyapatite, PEG, and anti-inflammatory agents to mitigate immune responses and promote tissue integration [43,50,102,127]. Hybrid designs and surface-engineered coatings, including electrosprayed PLGA films, hold promise for improving implant–host interactions and reducing inflammation or thrombosis [102].

Multifunctional systems are also gaining momentum, combining therapeutic and diagnostic capabilities. For instance, in situ PLGA-PDMS-forming implants co-loaded with rosuvastatin and copper–selenium nanoparticles have shown dual antimicrobial and cytotoxic effects in breast cancer models, highlighting the potential of integrated therapy platforms [43]. PLGA-PEG triblock copolymers offer enhanced release control for sensitive molecules like naltrexone [127], while PLGA-embedded gelatin sponges show promise for targeted lymphatic drug delivery and metastatic disease control [73].

Predictive modeling and advanced in vitro platforms are becoming essential tools for implant development. Finite element modeling (FEM) is increasingly used to simulate drug diffusion and degradation in nanofiber or hydrogel systems, enabling rational design and formulation optimization [136]. Gel-based platforms now better replicate subcutaneous environments and allow for real-time pH monitoring and accelerated release modeling, supporting in vitro–in vivo correlation (IVIVC) and regulatory translation [93,114]. Non-invasive modalities such as benchtop MRI and EPR spectroscopy offer continuous in vivo tracking of implant behavior [93], while release profiling techniques like the “tubule” sample-separate method show strong IVIVC (R^2^ > 0.99), facilitating robust quality control [155].

Clinically, PLGA-based systems are expected to play an increasingly central role in treating chronic conditions, rare diseases, and hormone-related disorders. Applications span long-term contraception, immunosuppression, antiangiogenic therapies, and site-specific oncology treatments [36,38,39,96,156]. The integration of bioactive components—such as dexamethasone-loaded hydrogels and PLGA/PHB nanocomposites—further supports their utility in regenerative medicine and orthopedics [137,144,150]. To fully realize this potential, future efforts must focus on large animal and clinical studies to evaluate long-term safety, biodegradation kinetics, and therapeutic efficacy [144,150,173]. Additionally, the convergence of PLGA with gene and cell therapies, as well as stimuli-responsive systems, is likely to define the next generation of implantable medical technologies.

## 11. Conclusions

PLGA-based implants represent a transformative convergence of materials engineering and biomedical science, delivering advanced solutions for controlled drug release, tissue repair, and disease-specific therapy. Their success lies in the tunability of polymer characteristics, the integration of bioactive agents, and innovative structural designs that collectively enable personalized and localized treatment strategies.

Although challenges persist—including burst release, degradation variability, and manufacturing complexity—recent advances in surface modification, predictive modeling, and multifunctional systems offer promising solutions. The development of hybrid materials, multilayered configurations, and computationally optimized implants marks a significant step forward in enhancing PLGA performance and expanding their clinical reach.

As testing methodologies and design technologies evolve, PLGA implants are positioned to set new standards in precision medicine, regenerative therapies, and chronic disease management. Future progress will depend on sustained interdisciplinary collaboration, rigorous translational research, and continued investment in scalable, patient-specific solutions. Ultimately, PLGA technology is poised to play a central role in the future of personalized healthcare, advancing long-term, targeted, and effective therapeutic platforms.

## Figures and Tables

**Table 1:** Impact of composition and additives on PLGA implant properties.

PLGA and Additives in Implants	Expanded Patterns in Applications	Refs.
PLGA + Glycerol, Ethyl Heptanoate	Modifies implant morphology to reduce burst release and enhance mechanical stability; effective in opioid addiction treatment and antibiotic delivery systems.	[56,57,58]
PLGA + Ibuprofen	Widely used in pain relief and inflammation therapy, with hot melt extrusion and 3D printing methods enabling personalized and controlled release profiles.	[4,8,25,59,60]
PLGA + Dimethyl Sulfoxide (DMSO)	Facilitates in situ forming depot systems, reducing burst release for psychiatric and hormone therapies; supports rapid implant solidification.	[55,61]
PLGA + PEG	Enhances protein and peptide release by neutralizing acidic degradation; also used as a plasticizer in implants for flexibility and tailored degradation rates.	[3,21,23,40]
PLGA + Albumin–Oleic Acid Conjugates (AOC)	Double-controlled release systems for psychiatric drug delivery, reducing burst effects and enabling sustained release over weeks.	[62]
PLGA + β-Cyclodextrin (β-CD)	Improves peptide delivery by modulating polymer erosion and sustained drug release; effective in cancer and hormone therapies.	[12,42,63]
PLGA + Hydroxyapatite or β-TCP	Promotes bone regeneration by enhancing osteoconductivity; used in dental, orthopedic, and spine applications with sustained release for months.	[45,64,65,66,67]
PLGA + Poloxamer, PEO	Adjusts polymer swelling to control drug release; ideal for local anesthetic delivery with tunable degradation and erosion properties.	[10]
PLGA + Dexamethasone	A key agent in inflammation control, delivering sustained release in neural, ocular, and implantable medical devices for weeks to months.	[22,37,40,68,69]
PLGA + Antibiotics (e.g., Ciprofloxacin, Gentamicin)	Provides localized infection control in orthopedic and dental implants; coatings offer sustained antibacterial activity and biofilm prevention.	[44,45,70]
PLGA + Metal Nanoparticles (e.g., Ag, Fe_3_O_4_)	Combines antibacterial and bioactive properties for implant coatings; effective in reducing infections and promoting osseointegration.	[44,45,71]
PLGA + Paclitaxel, Doxorubicin (DOX)	Used in advanced chemotherapy delivery systems, integrating phase-specific release for targeted tumor reduction and minimal systemic toxicity.	[6,27,72,73,74]
PLGA + Trehalose, Chitosan	Enhances protein stability and biocompatibility; chitosan contributes to tissue integration in neural, ocular, and bone repair systems.	[48,75,76]
PLGA + Various Solvents (e.g., NMP, TEC, Ethanol)	Solvent systems influence implant morphology, burst release, and sustained delivery; commonly used in in situ forming implants and depot formulations.	[9,23,54,56,58]
PLGA + Alginate or Gelatin	Enhances structural support and drug retention for cartilage, neural, and bone regeneration; supports dual-release systems for complex treatments.	[51,73,77,78]
PLGA + VEGF or Growth Factors	Stimulates vascularization and tissue repair, particularly in stroke and regenerative therapies; biphasic release supports long-term recovery.	[28,34,36,79]
PLGA + Fluorescent Markers or Dyes	Enables real-time imaging and monitoring of drug release profiles and implant degradation in vivo, supporting formulation refinement.	[18,80]
PLGA + Excipients (e.g., HPMC, Stearic Acid)	Modulates release profiles by altering implant swelling and phase separation; effective in anti-inflammatory and antibiotic delivery systems.	[23,41,81]

**Table 2:** Critical physicochemical traits influencing PLGA implant performance.

Physicochemical Property	Expanded Observations and Patterns	Refs.
Entrapment Efficiency	High efficiency observed (47.03–95.34%) across multiple applications. Factors include polymer compatibility, drug hydrophilicity, and particle size. For proteins/peptides, additives like PEG improved encapsulation and preserved bioactivity.	[1,61,86,96,97]
Drug Release Kinetics	Bi-phasic, tri-phasic, and near-zero-order patterns linked to polymer degradation and diffusion mechanisms. Additives and solvent choices significantly modified release phases, enabling longer release durations (up to 6 months for some formulations).	[4,19,21,22,68]
Burst Release	Reduced burst release (<5%) achieved through techniques such as altering solvent miscibility, adding PEG, or modifying polymer end groups. Formulations with medium MW PLGA (e.g., 34 kDa) and additives like shellac showed effective burst suppression.	[3,12,23,83]
Swelling Behavior	Swelling increased implant size significantly (600–1700%), promoting drug release by enhancing water penetration. Hydrophobic additives reduced swelling effects, creating more controlled release profiles.	[5,21,22,54,68]
Degradation Rate	Degradation onset (3–14 days) controlled by PLGA MW, lactide/glycolide ratio, and terminal group composition. Low MW and 50:50 ratios accelerated degradation, while higher lactide ratios slowed polymer erosion.	[4,5,14,98,99]
Morphology	Dense, porous, and sponge-like morphologies affected drug release and stability. Porous structures allowed faster release and better protein encapsulation, while dense morphologies delayed erosion and prolonged release.	[57,88,91,100,101]
Particle Size	Smaller particle sizes (~100 nm) favored rapid release and clearance, while larger particles (~10 μm) enabled prolonged drug delivery. Morphological transitions during release contributed to changing surface areas and release rates.	[3,48,73,96]
Controlled Porosity	Optimized pore sizes (300–500 μm) improved tissue integration and drug delivery, especially in bone regeneration. Excess porosity led to burst effects, while controlled pore architecture enabled sustained release.	[25,32,95]
Glass Transition Temperature (Tg)	Lower Tg (<20 °C) allowed flexible implant fabrication, critical for applications like injectable systems and neural implants. Tg affected mechanical properties and degradation rates.	[19,82,84]
Surface Modification	Surface coatings (e.g., plasma treatment, nanosilver, or bioactive glass) enhanced implant biocompatibility, corrosion resistance, and drug release. Modified surfaces also reduced bacterial adhesion and inflammatory responses.	[66,71,102,103]
Encapsulation Efficiency (Proteins/Peptides)	Protein formulations achieved encapsulation efficiencies >90%. Incorporating excipients like PEG improved protein stability and reduced denaturation, enabling effective long-term delivery.	[24,53,86,104]
Release Models	Korsmeyer–Peppas, Weibull, and Higuchi models effectively described release kinetics. Multi-phase models were common, reflecting the combined effects of diffusion, swelling, and polymer erosion.	[6,12,19,105,106]
Polymer Molecular Weight (MW)	MW directly influenced drug release and degradation. Medium MW (~34 kDa) associated with balanced release profiles; high MW (>50 kDa) delayed onset of degradation.	[5,7,22,101]
Lactide/Glycolide Ratio	Ratios of 75:25 provided slower, more sustained release profiles, ideal for depot formulations. Ratios of 50:50 favored faster degradation and burst effects, suitable for short-term therapies.	[2,20,64]
Surface Area to Volume Ratio	Higher surface-to-volume ratios accelerated release, with shape-controlled implants (e.g., honeycomb designs) demonstrating predictable release kinetics.	[17,84,107]
Residual Monomers	Residual monomer content increased degradation rates but reduced initial lag phases. Suitable for applications requiring rapid release initiation.	[19,30]
Hydrophilicity/Hydrophobicity	Hydrophobic additives (e.g., Eudragit S100) delayed drug release and reduced burst effects. Hydrophilic components (e.g., PEG) enhanced swelling and initial release.	[23,24,88]
Zeta Potential	Stable nanoparticle systems demonstrated zeta potentials around −30 mV, enabling efficient drug encapsulation and sustained release.	[108]
Drug Loading	High drug loading (>30%) accelerated burst release, requiring balancing with polymer additives to maintain controlled kinetics.	[12,88,96,109]
Diffusion-Controlled Release	Diffusion mechanisms dominated during early release phases, particularly in systems with large surface areas or controlled porosity.	[4,8,11,12]
Erosion Profiles	Implants exhibited bulk erosion in early phases and transitioned to surface erosion as structural integrity diminished. First-order kinetics commonly describes this behavior.	[54,68,98,104]
Elastic Modulus and Mechanical Properties	Mechanical properties decreased significantly as polymer degraded, with elastic modulus changes aligning with drug release and structural erosion.	[66,99,110,111]
Water Uptake	Higher water uptake correlated with rapid polymer swelling and enhanced drug diffusion. Formulations with hydrophobic additives exhibited delayed water absorption and prolonged release.	[22,85,93]
Biocompatibility	Additives like PEG, HPMC, and surface coatings reduced inflammatory responses. Implants demonstrated minimal toxicity in vivo, preserving tissue structure and function.	[50,102,104,112]

**Table 3:** Manufacturing techniques and their role in PLGA implant design.

Processing Method	Key Insights and Observed Patterns	Technical Data	Refs.
Hot Melt Extrusion (HME)	Stable implants; controls polymer-drug interactions and implant stability.	Temp: 90–120 °C; Pressure: 20–50 MPa; Drug–polymer blends milled before extrusion; compatible with heat-stable drugs.	[4,11,19,22,30,59,85,100]
Solvent Casting and Evaporation	Produces thin films and multilayer structures; controls burst release.	Solvents: Dichloromethane (DCM), acetone; Evaporation under reduced pressure; Thickness: 50–500 μm.	[12,101,121,122,123]
Phase Separation/Coacervation	Encapsulates hydrophilic and hydrophobic drugs; achieves controlled release.	Solvent: DCM, NMP; Non-solvent: Mineral oil, ethanol; Stirring rate: 200–1000 rpm; Particle sizes: 1–100 μm.	[15,82,124]
3D Printing	Enables tunable porosity and implant geometry.	Techniques: Fused Deposition Modeling (FDM), Direct Ink Writing (DIW); Print temp: 150–200 °C; Layer height: 0.1–0.5 mm.	[17,28,32,59,106]
Spray Drying	Produces uniform microspheres; enables controlled morphology.	Inlet temp: 40–80 °C; Solvent: Ethanol/DCM; Feed rate: 2–10 mL/min; Particle size: 1–10 μm; Encapsulation efficiency: 60–95%.	[3,73,107]
Micromolding and Compression c	Creates precise implant geometries; reduces burst release.	Compression force: 0.5–5 kN; Molding temp: 50–70 °C; Mold sizes: 1–10 mm; Drug loading: 10–50%.	[30,55,76,100]
Emulsion Techniques (o/w, w/o/w)	Produces microspheres and nanospheres with sustained release.	Solvents: DCM, acetone; Stabilizers: PVA, surfactants; Mixing speed: 500–1500 rpm; Encapsulation efficiency: 70–90%; Size: 200 nm–10 μm.	[27,36,48,125,126]
Solvent Exchange/Precipitation	Forms in situ implants with adjustable release profiles.	Solvents: NMP, DMSO; Non-solvent: PBS or aqueous buffers; Solvent exchange controlled by injection speed (1–5 mL/min); Morphology: Porous or dense structures.	[2,12,23,54,56,127]
Electrospinning	Produces nanofibrous scaffolds with high surface area.	Voltage: 10–20 kV; Feed rate: 0.5–2 mL/h; Solvents: DCM/DMF blends; Fiber diameters: 50–500 nm.	[60,128,129]
Microparticle and Nanoparticle Preparation	Enables stability for sensitive drugs; achieves extended release.	Techniques: Solvent evaporation, emulsification; Particle sizes: 100 nm–10 μm; Encapsulation efficiency: 70–95%.	[3,24,48,130]
Freeze-Drying (Lyophilization)	Preserves drug stability and bioactivity.	Freezing temp: −20 to −80 °C; Vacuum: 0.1–0.3 mbar; Drying temp: 0–25 °C; Process time: 24–72 h.	[107,125]
Microlithography	Creates precise microstructures; supports linear release.	UV-LIGA method; Resolution: 1–50 μm; Materials: PLGA 502, photoresist masks; Aspect ratio: Up to 20:1.	[120,131]
In Situ Phase Inversion	Injectable depots; solvent and polymer selection critical for performance.	Solvent: NMP, DMSO; Precipitation medium: PBS; Polymer concentration: 10–30%; Pore sizes: 1–100 μm.	[9,56,58,88,127]
Oil/Water Emulsion Solvent Evaporation	Produces uniform microspheres; scalable manufacturing.	Solvent: DCM, acetone; Emulsifiers: PVA, PEG; Stirring: 500–1500 rpm; Microsphere sizes: 500 nm–10 μm.	[40,42,48,116,123]
Ultrasonic Spray Coating	Applies uniform coatings for medical devices.	Ultrasonic frequency: 20–60 kHz; Solvents: Acetone, ethanol; Coating thickness: 10–100 μm.	[94,97,102]
High-Energy Ball Milling	Prepares uniform platforms with bioactive components.	Rotation speed: 200–400 rpm; Milling time: 2–8 h; Ball-to-powder ratio: 10:1–20:1; Size: 10–50 μm.	[99,132]
Hybrid Techniques (e.g., UMAOH)	Creates multifunctional implant coatings.	Process temp: 200–400 °C; Coating thickness: 10–50 μm; Hybrid layers: PLGA/CaP or PLGA/metal nanoparticles.	[66,133]
Multivariate Optimization Approaches	Adjusts parameters to minimize burst release and optimize drug loading.	Box–Behnken designs; Variables: Drug–polymer ratio, solvent composition, stirring speed; Results: Optimized burst control and morphology.	[55,57,127,134]
USP Apparatus 4 Testing	Accelerates implant release testing; correlates real-time data.	Testing medium: PBS; Flow rate: 1–10 mL/min; Temp: 37 °C; Testing duration: 1–60 days.	[135]
Mathematical and Computational Modeling	Predicts release kinetics; enables optimization of implant performance.	Finite element analysis; Models: Korsmeyer–Peppas, Higuchi; Inputs: Polymer properties, drug diffusion constants.	[18,80,136]

**Table 4:** Validation methods for PLGA implant safety and efficacy.

Test Type and Purpose	Key Observations	Practical Insights	Refs.
In Vitro Release: Assesses release kinetics, burst effects, and sustained release using HPLC, UV-Vis, etc.	Controlled release influenced by polymer type, molecular weight, and excipients.	Facilitates development of sustained delivery formulations for long-term therapies, reducing frequent dosing.	[1,9,61]
Morphological Analysis: SEM, TEM, and XRD to study surface properties, porosity, and internal structure.	Porosity and morphology directly affect drug diffusion and polymer degradation.	Guides design of implants with controlled release rates and predictable degradation behavior.	[4,63,82]
Degradation and Erosion Studies: Monitors mass loss, pH changes, and polymer erosion timelines.	Polymer degradation timelines vary with composition, pH environment, and drug loading.	Enables customization of polymer blends for targeted degradation profiles aligned with therapeutic needs.	[22,25,85]
Biocompatibility and Safety: Histological evaluations, inflammatory markers, and cytotoxicity assays.	Low inflammatory responses observed for optimized formulations; reduced toxicity in vivo.	Confirms safety and acceptance for clinical applications while minimizing adverse reactions.	[50,104,154]
Pharmacokinetics (PK): Monitors drug absorption, bioavailability, and systemic exposure in animal models.	Sustained therapeutic drug levels achieved with minimal burst effects.	Verifies prolonged action and reduces systemic toxicity, enabling better compliance in chronic treatment regimens.	[26,155,156]
Mechanical and Stability Testing: Evaluates tensile strength, viscoelasticity, and structural integrity.	High mechanical stability correlates with effective implantation in load-bearing environments.	Provides data for designing robust implants suitable for both load-bearing and soft tissue applications.	[49,99,111]
Toxicology and Immunogenicity: Quantifies immune reactions and long-term toxicity using animal models.	Minimal immune response with biodegradable polymers; compatibility varies with additives.	Supports formulation strategies that prioritize both safety and efficacy, particularly for sensitive or repeated use environments.	[50,66,89]
Advanced Imaging and Modeling: MRI, fluorescence imaging, and finite element analysis for in situ behavior.	Imaging revealed real-time degradation, swelling behavior, and release patterns.	Enhances prediction of in vivo performance, enabling better control over therapeutic outcomes.	[74,93,132]
Formulation Optimization: Designs tested using factorial designs like Box–Behnken for systematic evaluation.	Statistical modeling linked drug release with injectability and solidification parameters.	Simplifies the optimization process for formulations with complex interactions between polymer and drug characteristics.	[20,55,138]
Drug Stability Testing: Evaluates drug integrity post-processing and during release using FTIR, DSC, and XRD.	Structural and functional stability retained for proteins and sensitive drugs like monoclonal antibodies.	Enables reliable therapeutic delivery without loss of bioactivity, critical for sensitive treatments like cancer and autoimmune therapies.	[42,125]
Antimicrobial Efficacy: In vitro and in vivo tests against pathogens to verify antimicrobial properties.	Sustained activity observed against S. aureus, E. coli, and MRSA; minimal bacterial adherence on implant surfaces.	Useful for designing implants with dual therapeutic and infection prevention roles, especially for orthopedic and dental applications.	[45,128]
Controlled Release Evaluation: Studies using diffusion kinetics, Korsmeyer–Peppas models, and Weibull fitting.	Biphasic and triphasic release profiles validated for hydrophilic and hydrophobic drugs.	Provides mechanistic insights to tailor implants for various therapeutic needs, including cancer, diabetes, and chronic pain management.	[14,121,122]
Histological and Micro-CT Analysis: Quantifies tissue integration and implant–host interactions.	Enhanced bone growth and soft tissue integration in optimized formulations; reduced inflammation.	Informs development of implants with improved biocompatibility and functional outcomes for musculoskeletal and dental applications.	[28,94]
Gene Expression and Cellular Studies: Evaluates regenerative capabilities through qPCR, histology, and immunohistochemistry.	Increased osteogenic and chondrogenic markers; enhanced tissue repair observed in scaffolds with bioactive factors.	Supports development of bioengineered implants for tissue regeneration applications in orthopedics and neurology.	[46,157]

**Table 5:** Advances in therapeutic and diagnostic benefits of PLGA implants.

Category	Expanded Proven Benefits of PLGA-Based Implants	Refs.
Controlled Drug Release	- Extended-release profiles: Achieves drug release up to 6 months, ensuring stable therapeutic effects for chronic diseases. - Phase-controlled delivery: Combines burst, diffusion, and degradation phases for tailored outcomes. - Mitigating release variability: Controlled release in biphasic or triphasic patterns reduces drug wastage and patient variability.	[4,6,16,68,101]
Enhanced Patient Compliance	- Minimizes dosing frequency: Offers quarterly or biannual treatment options for conditions like prostate cancer, schizophrenia, and diabetes. - Simplifies therapy: Injectable formulations eliminate the need for daily pills or hospital stays. - Convenience and adherence: Long-term implants (90+ days) improve adherence for patients with complex therapeutic regimens like chemotherapy or HIV treatment.	[64,109,127,164,169]
Targeted Therapeutic Effects	- Local delivery systems: High drug concentrations at target sites reduce systemic exposure and toxicity (e.g., in cancer and ocular diseases). - Enhanced efficacy: Tumor shrinkage (>70%) and effective bacterial eradication demonstrated in preclinical and clinical studies. - Synergistic treatments: Combines effects like magnetic hyperthermia and localized chemotherapy for better outcomes.	[2,74,113,125,165]
Biocompatibility and Safety	- Low immunogenicity: Biodegradable and well tolerated even in sensitive tissues like the brain and eyes. - Adaptable formulations: Additives like PEG and HPMC improve compatibility, reducing adverse reactions in post-surgical or inflammatory conditions. - Environmentally sustainable: Biodegradation reduces long-term polymer accumulation, avoiding foreign body reactions in 90%+ cases.	[22,29,50,141,170]
Versatility in Drug Delivery	- Broad drug compatibility: Effectively delivers antibiotics, anticancer agents, analgesics, and hormones. - Platform versatility: Supports solids, liquids, and nanoparticle-encapsulated drugs. - Cross-domain applications: Demonstrated success in therapeutic areas such as oncology, orthopedics, ophthalmology, and endocrinology.	[31,42,134,137,171]
Surgical and Non-Surgical Use	- Reduced invasiveness: In situ forming systems eliminate surgical implantation for numerous applications. - Customizability: Designed for unique clinical needs, from injectable ISFIs to hybrid scaffolds for regeneration. - Cost-efficient alternatives: Minimizes surgery costs with bioresorbable options, avoiding secondary removal procedures.	[32,83,84,117,167]
Reduced Environmental Impact	- Degradable components: Breaks down into lactic and glycolic acid, which are easily metabolized. - Biopolymer blends: Optimized degradation rates ensure compatibility with environmental standards in biomedical waste. - Minimizes waste burden: Bio-resorption avoids post-treatment disposal requirements for implants.	[19,49,66,98,172]
Stability and Protein Retention	- Retains bioactivity: Encapsulated proteins (e.g., growth factors, monoclonal antibodies) remain active for weeks, critical for neural and vascular regeneration. - Avoids denaturation: Advanced stabilization techniques preserve sensitive drug integrity under various conditions.	[34,42,53,85,173]
Improved Pharmacokinetics	- Stable plasma levels: Reduces peaks and troughs for drugs with narrow therapeutic windows. - Optimized bioavailability: Achieves comparable or superior bioavailability to commercial formulations (e.g., Vivitrol^®^). - Tailored half-life management: Prolongs systemic effects of drugs while reducing off-target interactions.	[55,56,96,155,156]
Customization Potential	- Precision manufacturing: 3D printing and hot melt extrusion enable implant designs for unique patient or disease needs. - Regulated degradation rates: Controlled by molecular weight, lactide/glycolide ratio, or additives. - Shape and size adaptability: Supports personalized dosing and anatomical fit for complex or rare conditions.	[94,105,106,111,116]
Anti-Infective Capabilities	- Prevention of biofilm formation: Coatings with antimicrobial agents (e.g., nanosilver, antibiotics) enhance implant sterility. - Effective for resistant pathogens: Sustained antibiotic release tackles MRSA, S. aureus, and E. coli in orthopedic and dental settings.	[44,45,90,124,126]
Facilitation of Tissue Regeneration	- Bone healing: Enhances bone formation (50–85%) in femoral and cranial defects, matching or exceeding gold standards. - Cartilage regeneration: Promotes hyaline cartilage regeneration (increased GAG and type II collagen) for joint and meniscus repair. - Neural repair: Supports neurogenesis and angiogenesis in Alzheimer’s and spinal cord injuries.	[79,89,129,148,149]
Theranostic Applications	- Integrated monitoring: Imaging-compatible implants (MRI, CT) provide real-time insights into implant degradation and drug release. - Multi-functionality: Combines therapeutic effects (e.g., tumor ablation) with diagnostic capabilities, enabling personalized interventions.	[37,72,74,93,132]
Cost-Effectiveness	- Reduced treatment costs: Lowers healthcare burden by decreasing the frequency of hospital visits or surgeries. - Efficient manufacturing: Scalable production methods like microfluidics and solvent evaporation ensure affordability without sacrificing quality.	[14,30,43,135,143]

**Table 6:** Therapeutic applications of PLGA implants across medical fields.

Application Area	Specific Purposes	Drug Examples	Refs.
Drug Delivery	Sustained release of small molecules, peptides, and proteins for chronic disease management; reduces dosing frequency and enhances compliance.	Entecavir, Naltrexone, Leuprolide, Insulin, Dexamethasone, Buprenorphine	[1,15,40,53,61,127]
Pain Management	Prolonged release of NSAIDs, local anesthetics, or opioids for chronic and postoperative pain; supports synergy between drugs for improved efficacy.	Ibuprofen, Bupivacaine, Hydromorphone, Biphalin, Ketoprofen	[4,10,139,143,174]
Antibiotic Delivery	Localized infection control; prevents biofilm formation and promotes antimicrobial activity for dental and orthopedic implants.	Ciprofloxacin, Gentamicin, Chlorhexidine, Amoxicillin	[44,45,70,87,124]
Anti-Inflammatory Treatments	Long-term suppression of implant-related inflammation; used in neural implants, ocular devices, and implantable medical systems.	Dexamethasone, Methotrexate, Bupivacaine, Vancomycin, Spiramyin, Mitomycin C	[22,37,40,68,146,175]
Cancer Therapy	Localized chemotherapy with reduced systemic toxicity; combines hyperthermia and drug release for tumor ablation.	Paclitaxel, Doxorubicin, 5-Fluorouracil, Cisplatin, Carboplatin	[6,27,43,72,73,74]
Neuroregeneration	Promotes nerve repair, reduces glial scarring, and enhances biocompatibility for neural implants; supports spinal cord injury recovery.	Minocycline, VEGF, Anti-Nogo receptor antibody	[89,118,151,176]
Ophthalmology	Sustained intraocular drug delivery for conditions like glaucoma, macular degeneration, and ocular infections; supports posterior eye applications.	Dexamethasone, Clindamycin, Tacrolimus, Spiramycin	[16,39]
Bone Regeneration and Repair	Osseointegration in orthopedic and dental implants; supports vascularization and defect repair with biodegradable scaffolds.	Hydroxyapatite, β-TCP, VEGF	[45,65,66,67]
Vascular and Stroke Therapies	Stimulates vascularization for tissue repair; supports neovascularization in stroke recovery and regenerative applications.	VEGF	[28,34,36,79]
Cartilage and Osteochondral Repair	Regenerates articular cartilage; combines scaffolds and therapeutic agents to enhance structural integration.	Lithium ions, IGF-1	[14,51,149,150]
Hormone Therapy	Provides long-acting hormone release for prostate cancer, hormonal deficiencies, and contraception; minimizes dosing intervals.	Leuprolide acetate, Risperidone, Levonorgestrel, Rilpivirine	[2,64,75,96,101,109]
Periodontal Applications	Localized delivery of antibiotics and anti-inflammatory agents to treat periodontitis; improves adherence and mechanical retention.	Minocycline, Chlorhexidine, Secnidazole, Doxycycline	[81,87,163,177]
Diabetes Management	Controlled release of hypoglycemic agents for consistent blood glucose control; minimizes peaks and troughs in drug levels.	Linagliptin, Exendin-4, Glimepiride	[48,138,156]
Tissue Engineering	Sustained release scaffolds for soft and hard tissue engineering; supports adipose, cartilage, and nerve tissue formation.	IGF-1, VEGF, BSA	[51,85,95,141,148]
Post-Surgical Infection Control	Prevents infections at surgical sites using localized antibiotic release; avoids systemic side effects and improves healing.	Amoxicillin, Gentamicin, Rifampicin	[45,58,90,97,154]

## Data Availability

Not applicable.
